# Engaging children and families in pediatric Health Research: a scoping review

**DOI:** 10.1186/s40900-019-0168-9

**Published:** 2019-11-04

**Authors:** Rachel Flynn, Sarah Walton, Shannon D. Scott

**Affiliations:** Faculty of Nursing, Level 3, Edmonton Clinic Health Academy, 11405 87 Avenue, University of Alberta, Edmonton, Alberta T6G 1C9 Canada

**Keywords:** Patient engagement, Pediatric, Health services research

## Abstract

**Aim:**

Patient engagement (PE) in pediatric health services research is challenging due to contextual factors such as busyness of parenting, work schedules, and diverse family structures. This scoping review seeks to comprehensively map current PE strategies with parents and families across existing published pediatric health research literature.

**Methods:**

We followed Arksey and O’Malley (2005) and Levac et al., (2010) six-stage scoping review process. We conducted the search strategy in Medline, Embase, CINAHL, and Psychinfo databases. Data were extracted from included articles; evidence tables were developed and narrative synthesis was completed.

**Results:**

Of 3925 retrieved records, seventeen articles were included in the review. Patient engagement primarily occurred through strategies such as advisory groups, meetings, focus groups and interviews. Strategies were used to engage patients at various levels, for different purposes (e.g., to inform, participate, consult, involve collaborate and/or lead). These strategies were also used at various stages of the research process. Navigating power differences, time and money were commonly reported challenges. Inconsistent terminology plagued (e.g., stakeholder engagement, consumer participation, patient and public involvement, participatory research) this body of literature and clarity is urgently needed.

**Conclusions:**

This review offers insights into current PE strategies used in pediatric health services research and offers insight for researchers considering employing PE in the future.

## Plain English summary

Involving patients in health research is shown to lead to more relevant findings, and improve services and outcomes by meeting patient needs and concerns. Involving patients in the research process creates the patient as an equal partner, where the research focuses on answering patient questions as opposed to only addressing researcher questions. In child health research there are challenges to accessing and involving children and their families in the research process. We do not know the best ways to involve this population in health research. Better involvement of children and their families in research can lead to more meaningful and relevant findings for patients. There is a need to map out the current approaches used to involve children and their families in health research literature.

We therefore looked for studies that described any approach of child and/or family engagement in any stage of the research process. We explored what type of engagement strategy was used and the impact of engaging patients and/or their families in the research process.

We found 17 studies that included patients and/or their families at different stages of the research process. Patients and families were most commonly involved in research through focus groups, meetings, interviews, and working groups. Further work is needed to test the success of these commonly reported strategies. Understanding which strategies work best will help guide researchers on how to effectively engage children and their families to guide research.

## Introduction

Patient engagement (PE) is a process where patients meaningfully and actively collaborate in the governance, priority setting, and conduct of research, as well as in summarizing, distributing, sharing, and applying its resulting knowledge [1]. Multiple terms are used to refer to engaging the public in the research process (e.g., end users, stakeholders, parents, caregivers). In this paper, we use the term patient as “anyone who has personally lived the experience of a health issue as well as their informal caregivers, including family and friends” [1].

The ultimate goal of engaging patients in the research process is to optimize healthcare systems and practices. Patients can provide valuable insight into how to improve recruitment, data collection and other research methods due to their specific perspective [2, 3]. PE views the patient as an equal partner and focuses on answering patient questions as opposed to only addressing researcher questions. PE can have a positive impact on researchers through creating a greater understanding and “insight” into their area of study [4]. Furthermore, it has been shown that patients who are involved in the research process gain more knowledge about their conditions and may feel empowered with new life skills [4]. PE aims to ensure accountability, transparency, relevancy, and the production of unique insights that may otherwise not be found in the research process [5]. Involving patients in the research process has shown to produce better outcomes [5].

Despite these recognized benefits, PE requires significant resources, specifically time and money [6] which unfortunately can pose major barriers to authentic and successful PE. Furthermore, a lack of preparation, training, and feedback for those engaging in the research process, poses additional challenges [4].

### Patient engagement in child health research

There is a paucity of literature that comprehensively synthesizes strategies for implementing PE in child health research. Investigation in this area is valuable because parents and their children are often difficult to access for research [7]. The busyness of parenting, work schedules, and diverse family structures are some obstacles. The latter, compounded by the unique challenges of child health (i.e., working with vulnerable populations, developmental challenges) and its ethical considerations, results in challenges to recruitment and engagement in the research process. There is a need to comprehensively map out the current strategies used for PE within the pediatric health research literature. In this paper we define child from birth to 18 years of age (or extended to 21 if stipulated by the study).

### Aim & objectives

We conducted a scoping review to identify, synthesize and present the current state of the science on PE in pediatric health research. Our scoping review was guided by the question: *What are the various strategies that have been used to engage patients and families in pediatric health research?* The objective of the review was to better inform and equip child research investigators on how to engage patients in their research process.

## Methods

### Design

We conducted a scoping review using the Arskey and O′ Malley framework [8] that includes a six-sage process and the most recent recommendations outlined by Levac et al. [9]. Scoping reviews are defined as exploratory projects that systematically map the literature available on a topic, identifying key concepts, theories, sources of evidence and gaps in the research [10].

### Stage 1: identifying the research question

The research question that guided our scoping review was: *What are the various strategies that have been used to engage patients and families in pediatric health research?* We used the Population, Intervention, Comparison and Outcome (PICO) framework to establish our population (paediatrics and/or parents/family) intervention (engagement in research), comparison (none) and outcome (impact of patient engagement) of interest [11].

### Stage 2: identifying relevant studies

Our search strategy (Additional file 1.) developed by a health sciences research librarian was applied to Medline, Embase, CINAHL, and Psychinfo databases. Only studies available in full-text English were included.

### Stage 3: study selection

Our inclusion criteria (Table 1) included the following: (1) studies must be primary research, (2) patients and/or their families worked with a research investigator or team and contributed to the research design and/or process, (3) engagement occurred with children ranging from birth to 18 years of age (extended to 21 if stipulated by the study) and/or their family, and (4) engagement occurred in the health research contexts which included any environment where acute care services or tertiary care had or were occurring. All study designs were included to capture the full capacity to which PE has been utilized.

**Table 1:** Inclusion and exclusion criteria

Inclusion criteria	Exclusion criteria
Primary research	Patients defined as passive participants in research (subjects) or active recipients of clinical care.
Study implements a form of PE as defined by CIHR	Engagement of community members or other public stakeholders alone (not including patients and their families).
Study is engaging past or present patients and/or their family ranging in age from birth to 18 (or extended to 21 if stipulated by the study).	Non-acute care settings like public health, for example
Health research context included any environment where acute care services or tertiary care had or were occurring	Community-based participatory research

**Fig. 1:**
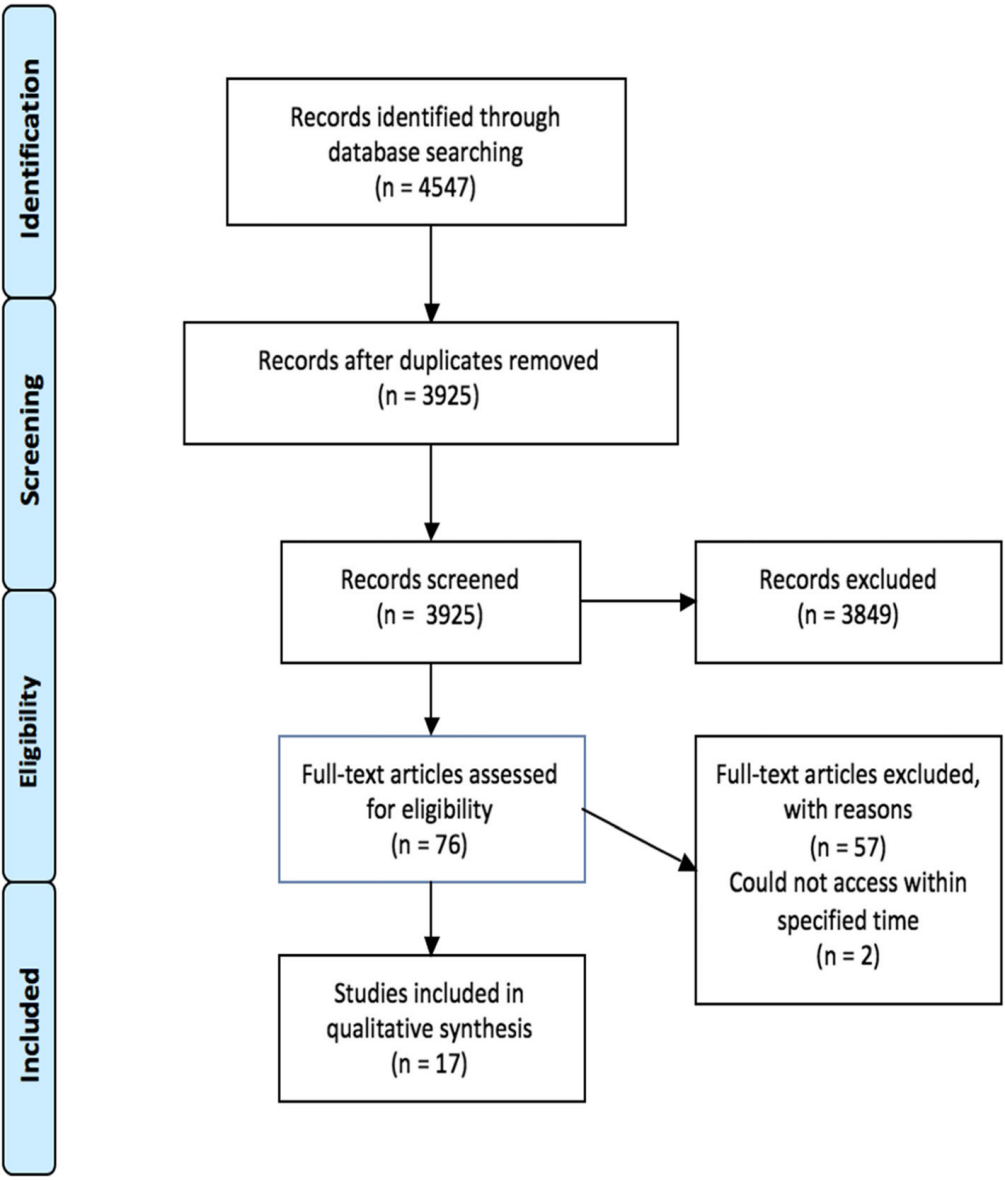
PRISMA flow diagram

Study selection occurred as a two-stage screening process with a primary and secondary reviewer (initials of two reviewers) who independently screened studies, to ensure inter-rater reliability. In the first stage of screening, (initials of primary reviewer) reviewed the titles and abstracts of all studies found from the four databases after duplicates were removed (*n* = 3925). The secondary reviewer (initials of secondary reviewer) independently reviewed 10 % of these titles and abstracts (*n* = 393) to assess screening reliability. Based on the inclusion and exclusion criteria, studies were divided into an “exclude,” “include,” or “unsure” category. During second stage screening, (initials of primary reviewer) screened the full-texts of all articles that were included from the first stage of screening (*n* = 76). (initials of secondary reviewer) screened a random 10% of the studies included in the second stage screening (*n* = 8). One hundred percent agreement was achieved. Two studies were not available in full-text format. We used Endnote X7 to manage data screening. Fig. 1. PRISMA flow diagram depicts our screening and selection process [12].

### Stage 4: charting the data

We extracted data on author(s), year of publication, country of origin, purpose, study design, study population and sample size, type of engagement strategy, effectiveness/impact of the PE on the research process, suggestions for future engagement or research, and any other relevant key findings from the seventeen included studies. Using these data we created an evidence table. To ensure the most appropriate information was being extracted, (initials of secondary reviewer) independently extracted data from a random10 percent of the final included studies (*n* = 2) using the data extraction form.

In order to classify the level of PE used in each study, we used an adapted version of the International Association of Public Participation (IAP2) levels of patient engagement spectrum, from the Alberta Strategies for Patient Oriented Research (SPOR) Support Unit [13]. The IAP2 spectrum classifies the levels of PE on a continuum from least to greatest time and knowledge needed for engagement. The continuum includes six different levels: learn/inform, participate, consult, involve, collaborate, and lead/support [13]. It is important to note that each included article was classified based on their own report of PE (or lack thereof). Also, an article could employ more than one level of PE and be represented on more than one level on the spectrum. In addition to level of PE, we also wanted to identify and map any patterns between the PE strategy selected (e.g., focus group) and the specific stages of childhood (e.g., neonate, infant) of those engaged in the research process. Therefore, we also extracted the different stages of childhood where reported by the authors of the included studies.

### Stage 5: collating, summarizing, and reporting results

Data analysis for the scoping review was descriptive. We collated and summarized characteristics of the included studies, such as study design, study population, and year of publication. We also collated and summarized where possible the benefits and challenges to PE reported in the included studies. Lastly, the meaning of the findings and their implications for future research, practice and policy were considered. Findings were drawn based on the synthesized results from stage four and five.

### Stage 6: consultation (optional)

Two consultation meetings with a local patient-orientated research expert group (Alberta SPOR Support Unit) occurred to request expertise, and feedback on the scoping review process [8, 9]. We received positive feedback on our study question and search strategy and an additional resource for extraction was provided (i.e.IAP2, adapted version).

## Results

### Study characteristics

The 17 included primary research studies [14–30] were published in the United States (*n* = 6) [14, 22, 25, 26, 28, 30], the United Kingdom (*n* = 5) [16, 19, 20, 23, 27], the Netherlands (*n* = 2) [21, 29], Canada (*n* = 1) [24], Australia (*n* = 2) [17, 18], and combined Canada and the United States (*n* = 1) [15]. The majority of the studies were published after 2010. Study design was classified based on how it was reported by the author in the article. The most commonly reported research designs were case studies (*n* = 7) [14, 15, 18, 21, 24, 28, 29], randomized control trials (*n* = 3) [16, 20, 27], qualitative design (*n* = 2) [22, 23], mixed-methods (*n* = 1) [19], multi-methods [25] comparative effectiveness research [26] and psychometric research [30]. The people engaged in the research process itself were most commonly a combination of parent and child (*n* = 5) [15, 16, 22, 26, 27] solely parent (*n* = 5) [14, 17, 19, 20, 28], family and child (*n* = 3) [18, 21, 24], solely family members (e.g., siblings) (*n* = 2) [23, 25], parent and family (*n* = 1) [30] and solely children or adolescents (*n* = 1) [29]. No patterns between the selected PE strategy and stage of childhood of those engaged were found. Characteristics of the included studies are reported in Table 2.

**Table 2:** Characteristics of the included studies

Author/Year/Country/ Reference	Study design	Setting	Engaged sample characteristics
Andonian, 2008, USA [14]	Case study	Mental health	Parents
Bartlett et al., 2016, Canada and USA [15]	Case Study	Rehabilitation medicine	Parent & child
Boote et al., 2016, UK [16]	Randomized control trial	Primary care	Parent & child
Byas et al., 2003, Australia [17a] [17b]	Naturalistic inquiry	Mental health	Parents
Curtin & Murtagh, 2007, Australia [18]	Case study	Occupational therapy (1) Motor impairment and (2) Acquired brain injury)	Family & child
Dixon-Woods et al., 2011, UK [19]	Mixed- methods	Maternal and child health	Parents
Edwards et al., 2011, UK [20]	Randomized control trial	Pediatric cerebral palsy	Parents
Elberse et al., 2011, Netherlands [21]	Case study	Congenital heart disease	Family & child
Luff et al., 2016, USA [22]	Qualitative	Pediatric chronic illness	Parent & child
Malcolm et al., 2008, UK [23]	Qualitative	Children’s hospice care	Family
Mongeau et al., 2007, Canada [24]	Case study	Pediatric palliative care	Family & child
Osher et al., 2001, USA [25]	Multi-methods	Mental health	Family
Saunders et al., 2016, USA [26]	Comparative effectiveness research	Adolescent cardiovascular health	Parent & child
Tume et al., 2016, UK [27]	Randomized control trial development	Pediatric intensive care unit	Parent & child
Uding et al., 2007, USA [28]	Case study	Pediatric chronic care	Parents
van Staa et al., 2010, Netherlands [29]	Case study	Pediatric chronic illness	Child
Wells et al., 2015, USA [30]	Psychometric research	Pediatric/youth special needs	Parents & family

### Terminology used and levels of engagement

Throughout the studies, different terms were used to describe the varying degrees and levels of patient and family engagement. Terms used included partnership, collaboration, consultation, participation, engagement, and involvement. Employing the adapted version of the IAP2 spectrum schematic [13], Table 3 describes the various levels of engagement employed. Only two articles [16, 18] reported engagement at the level of “lead/support.” The majority of articles reported engagement at the “consult” level (*n* = 10) , [15–17, 19, 21, 23, 25, 27, 28, 30], and the “involve” level (*n* = 9) [14–16, 18, 22, 25, 26, 28, 29].

**Table 3:** Level of patient engagement in research

Level of patient engagement	Articles employing levels of patient engagement	Description of level (based on IAP2 adapted version by SPOR)
Learn/Inform	16, 22, 26, 29	“In open atmosphere for sharing through orientation and information sessions, and media campaigns”
Participate	18, 20,23, 24, 26, 27, 28	“Through quantitative, qualitative, or mixed methods research”
Consult	15, 16, 17a, 19, 21,23,25, 27, 28, 30	“Through scientific cafes, focus groups, priority settings activities, and as members of ad hoc working groups or expert panels”
Involve	14, 15, 16, 18, 22, 25, 26, 28, 29	“As members of standing working groups and advisory committees or panels”
Collaborate	14, 17b, 24, 28, 29	“Patients as co-investigators”
Lead/Support	16, 18	“Through patient or community steering committees and patients as principle investigators”

### Patient engagement strategies

Researchers employed a wide variety of PE strategies. Focus groups [17, 19, 23, 25, 27, 28], advisory groups [16, 22, 26], interviews (both in person and on the phone) [17, 18, 20, 21, 23, 24, 26, 29] and meetings [14, 15, 20, 21, 24, 26, 30] were the main strategies for how PE was incorporated into the research process. In one study PE also occurred through feedback surveys [27]. Patients and/or their family were also reported as acting in the role of a co-researcher or co-investigator [15, 17b, 24, 28, 29] Where it was not feasible to interact in a face-to-face environment, webinars and teleconferencing technologies were used to garner involvement from a larger population and span wide geographical spaces [15, 26]. Training or orientation for the engaged population was reported in four articles [14, 22, 26, 29]. Less common strategies for PE occurred via steering committees [16, 18], social events [22, 24] and surveys [23, 26].

### Research stage where patient engagement occurred

PE activities were reported over the entire research process. Engagement in research agenda/priority setting [21, 23, 27], research design [16, 20, 22, 26, 28, 30] and recruitment [15–18] were common. Other activities that patients and/or their families were involved with include developing focus group, interview, and questionnaire content as well as reviewing and refining the layout and wording of various documents throughout the research process [17, 27, 29]. In four studies PE occurred in data collection, analysis, and interpretation [14, 19, 24–26, 29]. PE occurred during a variety of research dissemination activities. For example, the engaged population presented at conferences [15, 17], contributed to publications [15, 17, 28], sent out emails or taught classes [19, 28, 29].

### Benefits and challenges of patient engagement

Table 4. outlines the benefits and challenges of PE in health services pediatric research. These benefits and challenges were explicitly reported by the authors of the included studies and not inferred by us (authors of the review). The benefits and challenges are divided up into those affecting the research itself and those affecting the engaged population.

**Table 4:** Benefits and challenges of patient and family engagement in pediatric research

	Benefits	Challenges
Research	• Creates sense of genuine value and purpose [14, 15, 17, 21, 22, 28] • Influenced and enhanced trial design [16, 20, 22, 25, 26, 30] • More relevant findings [17, 18, 20, 24, 26] • Increased recruitment and retention rates [20, 21] • Enhanced public exposure broadened dissemination and social relevancy [19, 24, 25, 28] • Increased accuracy and/or utilization of results [24, 25] • Families “revitalized” and “motivated” the researchers [22, 26]	• High investment of time and money [15, 16, 28, 29] • Recruitment of individuals for PE [15, 16, 27] • Sustaining engagement [23, 29] • Achieving representative and diverse engaged population [15, 18] • Merging and representing contrary and varying experiences from parents [18, 23, 28, 30]
Engaged population	• Felt empowered and/or able to “give back” [21, 24, 26, 28, 29] • Increased motivation, awareness, and confidence [14, 22, 28, 29] • Gained new knowledge and skills [22, 28, 29] • Form of therapy for bereaved families [23]	• Equalizing power imbalance between researcher and patient [15, 18, 24, 26] • Navigating logistics (sick children, family schedules, geographical distances) [23, 24, 26–28] • Lack of knowledge (medical jargon, research process) [14, 21, 23, 24, 26] • Establishing relationships and trust [24, 26] • Involving and/or maintaining children and family in higher levels of PE [25, 29]

## Discussion

We mapped the PE research in child health. Other previous works have identified similar common PE strategies across different health research contexts and at different stages of the research process. For example, Boote et al. [31] also found that meetings were one of the most common PE strategies employed for research. Similarly, focus groups, interviews and surveys have been reported as common strategies to engage patients in the research process [32]. It has been recognized in previous work that the PE strategies employed should be tailored to the research topic [33–35]. Another systematic review identified important factors to be considered during the pursuit of PE in the research process, such as providing education and training to the engaged population, ensuring the engaged population play an active role where roles, expectations and goals are set [36]. Our scoping review found that despite the use of training as a PE strategy, lack of knowledge in the research process was a reported barrier to successful PE [14, 21, 23, 24, 26]. Those embarking in PE research need to consider the purpose and content of the training to ensure it increases patient’s knowledge in the research process.

When engagement occurred in the form of co-researchers or co-investigators , [14, 17, 24, 28, 29], the engagement was much more time-consuming, required more education and expertise, and often included some form of stipend or honorarium to the patient or family member. Brett et al. [37] found that PE throughout the entire research process may have a more positive impact. Further research is needed to examine this. Many of the challenges described in our review have been reported in previous reviews. For example, Domecq et al. [3] reported time constraints and funding required to continue PE as barriers to engagement. They also reported concerns of researchers that PE may become tokenistic and that patients may present issues relevant to them but not to research [3]. A recent study by Taylor et al. (2018) also reported common challenges to child PE such as cost, life stage commitments, transition to adulthood and having a representation of the population [38].

Themes around navigating power differences between researchers and the “guest” researchers were shown [15, 18, 24, 28]. One study explained this concept by saying that children “have difficulty accepting an adult as non-directive and collaborative. Adolescents tend to lack experience of adults as participatory, enjoyable and non-judgemental” [18]. This therefore acts as a barrier for children to share their unfiltered and seemingly less educated opinions. The former combined with the social norm that adults “know best” [32, 39] may supress child input in the research process.

Our scoping review found that team meetings was one PE strategy which facilitated positive relationships between the researcher and patient population, built authentic partnerships and emphasized common shared values [14, 15]. Building trusting relationships, having a flexible approach by the researcher and placing the patient in a position of expertise were reported factors to mitigate the challenge of equalizing power balances in PE child health research [15, 18]. No significant relationship between level of involvement and age of child was shown. Not surprisingly though, parents were almost always involved in the collaborative and lead/support levels of patient engagement.

We also identified benefits to PE in child health research. When families and patients were engaged in the research process they felt empowered more confident, and enthusiastic [21, 24, 29]. A previous systematic review on PE, identified that is important for consumers that their involvement is valued by the researcher and that they are encouraged and given the confidence to contribute to changes in the research process [37]. Similar to the benefits of PE that we identified, a recent study on PE of young people with cancer also found that engagement in the research process provided them with new skills that they may have missed out on during treatment for their illness and also provided them the opportunity to see that a diagnosis does not put a stop to continuing their lives [38]. We consistently identified that PE in the research process led to more relevant and applicable research findings. Recruitment rates were higher, design elements improve, and dissemination methods were tailored to the needs of their specific populations. Brett et al. [37] also found that PE in both health and social research processes provided greater relevance and quality of the findings.

Although PE is becoming more commonplace in health research, it can be viewed as no more than a mandatory (and often trivial) requirement that must be checked off the research proposal list in order to receive funding [2]. Instead, a shift in thinking about PE must occur. Researchers must realize that PE is more than having, for example, a symbolic patient advisory committee providing input at a few points during the project. PE is a means to which the research process and subsequent findings can become more relevant and applicable to the community, thus bridging the gap between research and clinical practice [2].

### Limitations

The level of research reporting in the included studies may have limited the PE descriptions. Additionally, there was no formal evaluation of the effectiveness of the PE strategies and their impact found in any of the included studies.

## Conclusion

There is a paucity of knowledge regarding PE in the research process in child health contexts. Our review maps the current evidence on PE strategies in child health. To date, the most commonly reported PE strategies include qualitative methods, such as focus groups, meetings, interviews, and working groups. Further work is needed to test the degrees of effectiveness of these most commonly reported PE strategies. It is also unknown which strategies are most effective for which purpose and at what stage of this research. For example, our review demonstrated that PE was most commonly reported at a consult level- which occurs typically through scientific cafes, focus groups, priority settings activities, and as members of ad hoc working groups or expert panels. However it remains unknown which PE strategy is most effective for which level of engagement. Our findings also show that there is no clear pattern or guidance on what strategies of involvement have been used at what stage of childhood. This is important to identify as the relevance and appropriateness of PE strategy may vary according to age of the child/patient. Developing such evidence can provide more rigorous guidance to child health researchers who want to engage patients in their research. Knowing what strategies are most appropriate and successful for what age group will help inform researchers on what strategies to use to engage children patients in their research process. Future research is also needed to explore the concept that ‘adults know best’ and how this influences children’s’ input in the research process. It is important to understand whether the child’s point of view is taken into account and to determine the impact of PE in child health research.

## Supplementary information

**Additional file 1.** MEDLINE Search Strategy

## Data Availability

All data from the literature synthesized during this study are included in this published article [and its supplementary information files].

## Abbreviations

IAP2: International Association of Public Participation
PE: Patient engagement
PICO: Population, Intervention, Comparison and Outcome
SPOR) Support Unit: Alberta Strategies for Patient Oriented Research
