# Supplementary material for: Engaging children and families in pediatric Health Research: a scoping review
Source: Res Involv Engagem. 2019 Nov 4;5:32. doi: 10.1186/s40900-019-0168-9 (PMC6827239; doi:10.1186/s40900-019-0168-9)
Supplement: Supplementary file 1 — Additional file 1. MEDLINE Search Strategy [file 40900_2019_168_MOESM1_ESM.docx]

**Appendix 1. MEDLINE Search Strategy**

Ovid Technologies, Inc. Email Service
------------------------------
Search for: 13 and 15
Results: 1

Database: Epub Ahead of Print, In-Process & Other Non-Indexed Citations, Ovid MEDLINE(R) Daily and Ovid MEDLINE(R) <1946
to Present>
Search Strategy:
--------------------------------------------------------------------------------
1     exp Consumer Participation/ (35165)

2     ((Patient* or famil* or parent* or caregiver* or stakeholder* or community) adj3 (Engag* or participation or
participatory or involvement or involving or oriented or orientated or collaborat*)).mp. (92441)

3     1 or 2 (104728)

4     (Child* or pediatric* or paediatric* or teen* or adolesc* or youth* or toddler* or infant* or neonate* or
newborn*).mp. (3680445)

5     (research or health technology assessment*).tw,kf. (1101338)

6     3 and 4 and 5 (5295)

7     (research or trial*).ti. and 6 (1274)

8     (advisory board* or advisory committee* or advisory group* or panel or workshop* or working collaborativ* or
partnership* or PCORI or SPOR).mp. (170577)

9     6 and 8 (647)
